# Optimization of One-Time Fertilization Scheme Achieved the Balance of Yield, Quality and Economic Benefits of Direct-Seeded Rice

**DOI:** 10.3390/plants12102047

**Published:** 2023-05-21

**Authors:** Shuang Cheng, Zhipeng Xing, Chao Tian, Wen’an Weng, Qun Hu, Hongcheng Zhang

**Affiliations:** Jiangsu Key Laboratory of Crop Cultivation and Physiology/Jiangsu Co-Innovation Center for Modern Production Technology of Grain Crops, Research Institute of Rice Industrial Engineering Technology, Yangzhou University, Yangzhou 225009, China; cs1692135738@163.com (S.C.); tc18082379307@163.com (C.T.); wengwenan@163.com (W.W.); huqun@yzu.edu.cn (Q.H.)

**Keywords:** controlled release nitrogen fertilizer, conventional urea, mechanized direct seeded, rice, benefits evaluation

## Abstract

There is limited information available to assess the impact of one-time fertilization on the yield, quality, and economic benefits of direct-seeded rice. This study reports the effects of three one-time fertilizer treatments (BBU1, BBU2, and BBU3) on the yield, quality, and economic benefits of direct-seeded rice, where controlled-release nitrogen (N) fertilizer (CRNF) provided 50%, 60%, and 70% of the total N (270 kg N ha^−1^), and the control treatment (CK) was a split application of conventional urea (CU). The results showed that the yield of direct-seeded rice decreased significantly (*p* < 0.05) with the increased application ratio of CRNF under one-time fertilization, which was mainly related to N accumulation between the heading time and maturity stages. Compared to CK, the one-time fertilization treatments (BBU1, BBU2, and BBU3) maintained high milling quality, with significantly reduced chalkiness (*p* < 0.05), which could be related to the slow rate of N release from the CRNF. In addition, the one-time fertilization treatments reduced the protein content and increased the amylose content of the milled rice, which significantly improved the eating quality (*p* < 0.05). Furthermore, there was no significant difference in yield and economic benefit between BBUI and CK (*p* > 0.05). Overall, CRNF replacing conventional urea with 50% total N could be helpful to reduce fertilization frequency, achieve high yield and high economic efficiency, and improve rice quality of direct-seeded rice under one-time fertilization.

## 1. Introduction

Direct-seeding rice is a popular agronomic practice in Asia, North America, and Europe, because it eliminates the labor-intensive steps of raising and transplanting rice seedlings [1,2,3]. The agricultural management of direct-seeded rice also tends to be more efficient in terms of mechanized tillage, sowing, and harvesting, allowing a larger land area to be cultivated with less labor [4]. However, direct-seeded rice is still primarily split-fertilized, particularly in China, where a base fertilizer and multiple topdressing are often required [5]. In addition, two or three supplemental fertilizations may be required in drained or irrigated fields, depending on seedling status [6]. Although split fertilization has been repeatedly shown to achieve higher grain yields [7,8], the application of multiple topdressings has also been implicated in the deterioration of the eating- and appearance- quality of milled rice [9,10]. In addition, although fertilization efficiency has been greatly improved by modern methods, such as the use of wheeled throwing machines, considerable amounts of labor and materials are still required to cultivate rice [11,12]. As a result, improving the rice quality and economic efficiency of direct-seeded rice by reducing the frequency of fertilizer application while achieving high grain yield is an urgent issue.

Recently, a one-time fertilization scheme utilizing controlled-release nitrogen fertilizer (CRNF) has been developed for rice [5,13]. Studies in transplanted rice have shown that this one-time fertilization program improves root growth [14], delays leaf senescence [5,15], and increases biomass accumulation [16,17] and grain yield [13,18]. Other studies have tested modified one-time fertilization programs for transplanted rice, including the use of long-lived and short-lived CRNFs and the combined use of CRNF and conventional urea [19,20]. However, less effort has been made to optimize the application ratio of CRNF to conventional urea to achieve a balance among yield, rice quality, and economic benefits of direct-seeded rice under one-time fertilization. Therefore, the study of one-time fertilization programs in direct-seeding rice will not only help to promote the widespread adoption of sustainable and efficient fertilization practices, but also contribute to the further development of direct-seeded rice.

In China, direct-seeded rice is mainly grown in rice-wheat (rapeseed) or double-cropping systems [21,22]. Due to the short interval between crop rotations and the long-term restriction on the use of small-horsepower farm equipment, direct-seeded rice fields are often poorly-leveled, resulting in uneven seed germination [21,23,24]. In addition, the residue from the previous crop is often concentrated at the soil surface due to shallow rotary tillage, making it difficult for the roots of rice seedlings to make direct contact with the soil [25,26]. Recently, the operational efficiency of direct-seeded rice cultivation has been improved through the adoption and use of high-powered, multifunctional, integrated machinery [4,27]. However, studies evaluating the effects of one-time fertilization on direct seeded rice under high quality tillage are limited. Here, we tested three combinations of CRNF and conventional urea under one-time fertilization and verified their effects on yield, quality, and economic efficiency of direct-seeded rice under high-quality tillage status. Our aim was to develop a one-time fertilization program for direct-seeded rice to facilitate its adoption and further development.

## 2. Results

### 2.1. Nitrogen Release Profile of CRNF

**Figure 1:**
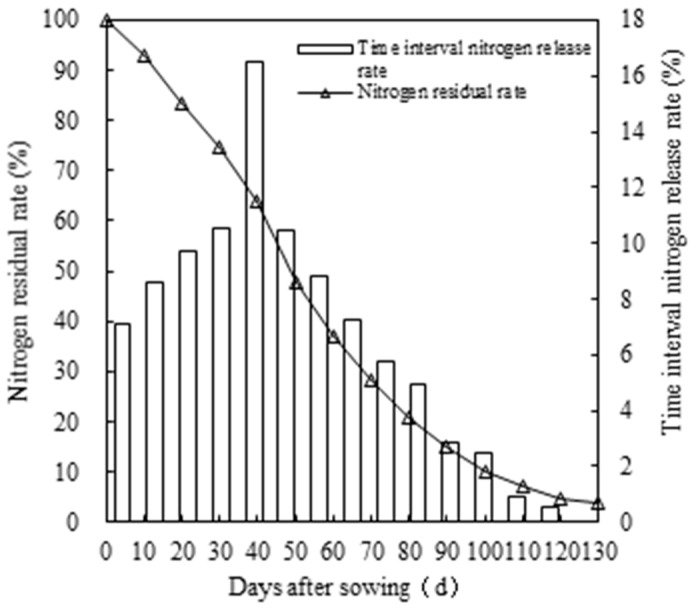
Nitrogen-release profile of controlled-release urea formula.

The N release profile of the CRNF exhibited a trend of progressively increasing then decreasing with time (Figure 1), with maximum N release occurring 40–50 days after bag burial. Because the N in the CRNF is released gradually over time, the N residual rate in CRNF was less than 20% at 80–90 days. These results indicate that the longevity of the tested CRNF was recorded as 80–90 days under the present experimental conditions.

### 2.2. Nitrogen Accumulation in Rice

**Figure 2:**
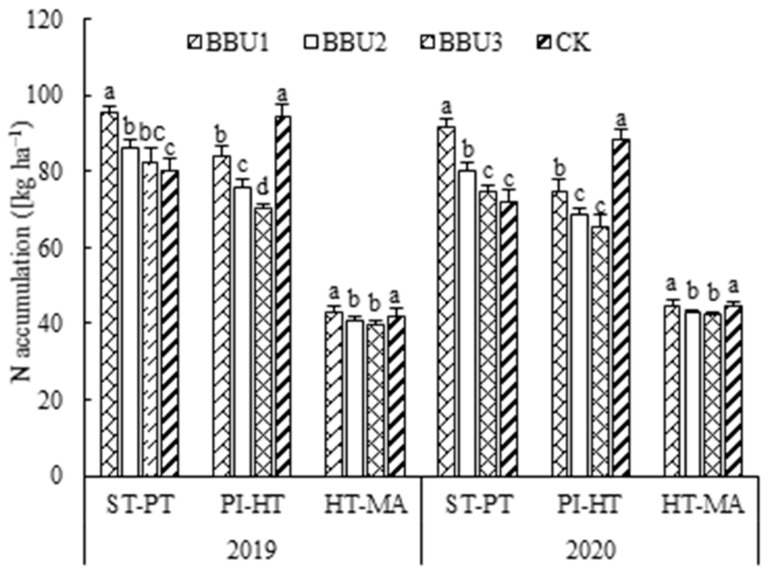
Effects of different treatments on the rice plants’ nitrogen accumulation. BBU1, controlled-release nitrogen fertilizer (CRNF), and conventional urea (CU) were base-applied at 50% of total N each; BBU2, CRNF and CU were base-applied at a 60% and 40% of total N, respectively; BBU3, CRNF and CU were base-applied at 70% and 30% of total N, respectively; CK, split application of CU. ST, PI, HT, and MA indicate sowing time, panicle initiation, heading time and maturity, respectively. The Vertical bars (mean values ± S.E.) with different letters in the same stage of the same year are significantly different (*p* < 0.05).

Different fertilization treatments exerted significant effects on N accumulation (Figure 2). At each growth stage, the BBU1 treatment resulted in the accumulation of significantly more N than either the BBU2 or BBU3 treatments. Additionally, from sowing time to panicle initiation stages, BBU1 accumulated 18.5–27.1% more nitrogen than CK; from panicle initiation to maturity stages, BBU1 and CK had no significant difference.

### 2.3. Photosynthetic Potential and Crop Growth Rate of Rice

The photosynthetic potential of rice leaves tended to decrease with increasing CRNF application in one-time fertilization (Table 1). Compared with the BBU2 and BBU3, the photosynthetic potential was 8.5–13.8%, 7.4–12.4%, and 8.2–15.3% higher in the BBUI from sowing time to panicle initiation stages, from panicle initiation to heading time stages, and from heading time to maturity stages, respectively. Compared to CK, the photosynthetic potential of rice plants subjected to one-time fertilization increased from sowing time to panicle initiation stages, and from panicle initiation to heading time stages; only BBU1 presented a higher photosynthetic potential than CK from heading time to maturity stages.

Different fertilization treatments had significant effects on the crop growth rate (Table 2). The crop growth rate of BBU1 was significantly higher than that of the other treatments at each stage under one-time fertilization. Compared with CK, the crop growth rate under one-time fertilization increased by 2.3–27.8% from the ST to PI, and decreased by 6.0–17.1% and 2.9–11.0% from PI to HT, and from HT to MA, respectively.

### 2.4. Rice Yield

**Figure 3:**
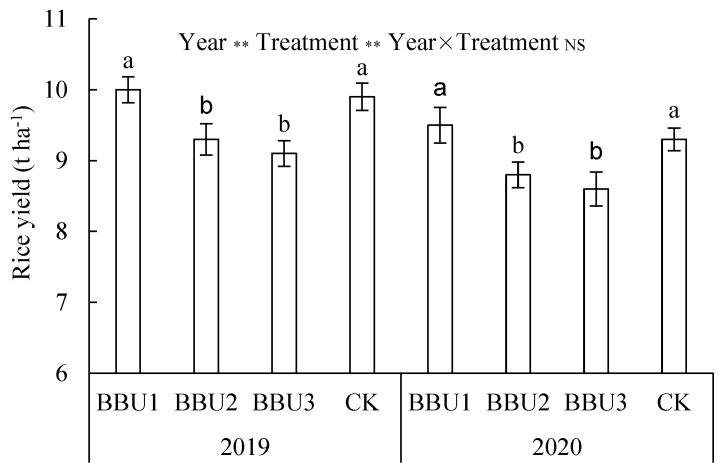
Effects of different treatments on the grain yield. BBU1, both controlled-release nitrogen fertilizer (CRNF) and conventional urea (CU) were base-applied at 50% of total N each; BBU2, CRNF, and CU were base-applied at a 60% and 40% of total N, respectively; BBU3, CRNF, and CU were base-applied at 70% and 30% of total N, respectively; CK, split application of CU. The vertical bars (mean values ± S.E.) with different letters in the same year are significantly different (*p* < 0.05).

Grain yield of direct-seeded rice tended to decrease with increasing CRNF application in the one-time fertilization treatments (Figure 3). The highest yields were achieved using BBU1 in both 2019 (10 t ha^−1^) and 2020 (9.5 t ha^−1^). Rice yields were 7.5–8.0% and 9.9–10.5% higher in BBU1 than in BBU2 and BBU3, respectively (*p* < 0.05). Rice yield was also higher in BBU1 than in CK, although the difference was not significant.

### 2.5. Milling Quality and Appearance Quality of Rice

CRNF reduction resulted in improved milling quality under one-time fertilization (Table 2), although neither year, treatment, nor their interactions significantly altered milling quality. The appearance quality of milled rice was also significantly affected by fertilizer treatment (Table 3). Compared to CK, one-time fertilization reduced the value of chalky parameters. Furthermore, each of these parameters tended to decrease with increasing CRNF application. Compared with CK, the chalky kernel rate, chalky area, and chalkiness of milled rice in the BBU3 treatment were reduced by 22.7–28.7%, 21.3–28.7%, and 43.3–44.4%, respectively (*p* < 0.05).

### 2.6. Contents of Amylose and Protein in Rice

Compared with CK, one-time fertilization increased the amylose content and decreased the protein content, especially the gliadin content, of milled rice (Table 3). Furthermore, the amylose content tended to increase, and the protein content tended to decrease with increasing application of CRNF. Compared with CK, the amylose content in the BBU3 was increased by 13.4–14.4%, the protein content was decreased by 9.7–11.1%, and the gliadin content was decreased by 14.3–18.7% (*p* < 0.05).

### 2.7. Rice Taste Quality

The rice taste value and its parameters were significantly affected by fertilization treatment (Table 4). One-time fertilization significantly improved rice taste value when compared with CK, which was further improved with increasing CRNF application. Compared with CK, the rice taste value increased by 17.3–18.2% in the BBU3. Additionally, the taste parameters of appearance, viscosity, and balance quality increased by 18.0–32.1%, 32.1–41.2%, and 25.935.3%, respectively, in the BBU3, (*p* < 0.05), while hardness decreased by 10.1–25.9% (*p* < 0.05).

### 2.8. Cost, Income, and Economic Benefit

The use of CRNF was associated with increased fertilizer costs compared to CK (Table 5). However, the increased fertilizer cost did not significantly increase the total production cost due to reduced fertilizer application. Compared to BBU2 and BBU3, BBU1 and CK achieved higher income and economic benefits due to higher grain yield.

## 3. Discussion

### 3.1. Effects of CRNF and CU Basal Application Ratios on Rice Grain Yield

BBU1 significantly increased rice yield in the three one-time fertilization treatments (Figure 3). This result could be attributed to several reasons. First, an appropriately increased N in the basal fertilizer could reduce the N competition between seedlings and wheat straw (high C/N ratio), thus mitigating the possible negative effects of wheat straw on seedling growth [21,28,29]. Second, the large amount of N release from conventional urea and CRNF in BBU1 0–50 days after sowing contributed to improved N uptake, tillering, leaf photosynthetic potential, and crop growth rate from sowing through panicle initiation [20,30,31]. Third, deep tillage (15~18 cm) not only effectively buried crop residues, leveled the soil surface, reduced soil capacity, and provided a healthy soil environment conducive to seedling growth [21,28], it also helped rice roots penetrate deeply into the soil and improved root uptake and utilization of deep soil nutrients [21,32,33]. Finally, increased N fertilizer inputs at the base fertilizer and rice tillering stages could promote the decomposition and fertilization potential of wheat straw and improve the nutrient availability of rice after heading [20]. It is worth noting that although efficient tillage has probably resulted in deep fertilization and reduced N loss during the early stages of rice growth [34,35], frequent precipitation events after basal fertilization in 2020 may have increased nitrogen release from CRNF, N loss and leaching [15], thereby reducing grain yield. Therefore, excessive rainfall is not conducive to the enhanced yield effect of one-time fertilization, and excessive basal applications of conventional urea (>50% of total N) in one-time fertilization are not recommended. Therefore, excessive rainfall is not conducive to the enhanced yield effect of one-time fertilization, and excessive basal applications of CU conventional urea (>50% of total N) are not recommended.

The optimized split-fertilization methods have been widely demonstrated to meet rice N demand after heading and to achieve high yields [36,37]. Consistent with the results of previous studies, we found that CK resulted in high N accumulation from heading to maturity stages and high yields. Recent reports have shown that one-time fertilization can increase N accumulation and grain yield in rice by better synchronizing N release with plant growth, compared to split fertilization [38,39]. In this study, we found that one-time fertilization did not significantly improve N accumulation and yield in this study, compared to CK. This result might be due to the fact that the N in the CRNF was not fully released at maturity (Figure 1), which might have reduced plant available N and decreased N accumulation and grain yield. In addition to the effects of coating material and moisture, N release from CRNF was significantly influenced by temperature [40,41]. Between 10–30 °C, the release rate of controlled-release fertilizer showed a non-linear increase with increasing temperature [42]. Huang et al. [5] reported that the N release rate of controlled-release fertilizer decreased significantly with decreasing temperature in a double cropping system. Therefore, efforts should be made to reduce the temperature sensitivity of CRNF, so that a one-time fertilization would be beneficial to both the complete release of N over the rice growth cycle and high grain yields.

### 3.2. Effects of CRNF and CU Basal Application Ratio on Appearance and Taste Quality of Rice

Chalkiness is an undesirable rice appearance trait, which is caused by the precipitation of starch and protein in the endosperm during rice filling, and is controlled by both genetics and agricultural management, including N fertilization [43,44]. Compared with split fertilization, one-time application of coated nitrogen fertilizer is helpful to improve the chalkiness characteristics of milled rice [38]. Similarly, we found that one-time fertilization reduced the chalkiness of milled rice compared to CK (Table 2). This result may be due to the fact that, compared with the rapid release of CU in panicle fertilizer, the stable release of nutrients from CRNF during the rice-filling stage facilitates a stable supply of N, promotes a favorable distribution of starch and protein in the rice grain, and reduces both the filling gap and light reflection, resulting in reduced chalkiness in the milled rice [18,24,38]. Several studies have reported that increased N accumulation during the filling period promotes a looser arrangement of starch granules in grains and increases chalkiness [45,46]. We found that, under one-time fertilization, the value of chalkiness parameters of milled rice tended to decrease with decreasing N uptake between heading and maturity (Figure 2, Table 2). This result might be due to the fact that reduced N accumulation during grain filling promotes the reactivation of non-structural carbohydrates, improves the development of amyloplasts, and reduces the dispersion of starch granules, which together reduce grain chalkiness [47,48].

Eating quality of milled rice is often considered to be the most important factor in determining consumer acceptance. In particular, amylose and protein contents are important factors in the eating quality of milled rice, both of which are controlled by carbon and N metabolism during rice growth [49,50]. Previous work has reported that one-time fertilization may reduce the protein content of milled rice, thus improving its eating quality [38]. This finding was mainly due to the fact that a high protein content is linked with an increase in hardness and a decrease in viscosity of the rice [51,52]. We also found that reduced protein content was associated with improved rice taste value under one-time fertilization. Studies have shown that reduced N accumulation during grain filling inhibits N metabolism, promotes more balanced carbon and N metabolism, reduces the protein content, and increases the amylose content, which together improve the eating quality of milled rice [31,48]. The protein in milled rice is mainly comprised of albumins, globulins, glutenins, and prolamins [51]. The contents of albumin and globulin are mainly under genetic control, while the contents of glutenin and prolamin can be significantly altered by N fertilization management [52,53]. It has been reported that fertilization at the panicle-initiation stage often results in an increase in the prolamin content, which reduces the viscosity of milled rice and limits the precipitation of starch during cooking, resulting in poor taste [38,54]. In this study, one-time fertilization reduced the gliadin and improved the taste value of rice compared with CK. This result indicated that the improvement of the eating quality by one-time fertilization may not only contribute to the reduction of the total protein content, but may also be due to the reduction of gliadin. However, although Nanjing 9108 is widely grown in eastern China and is well-known for its superior taste, the eating quality of different rice cultivars can vary widely due to genetic differences [55,56]. Therefore, further research should be conducted to study the effect of one-time fertilization on taste properties of other rice cultivars.

### 3.3. Effects of CRNF and CU Basal Application Ratios on Economic Benefits

For farmers, economic efficiency is one of the strongest drivers for adoption of agricultural management practices [57,58]. Partial replacement of CU with CRNF has been reported to be more economically beneficial than either split application of CU or full application of CRNF [39]. We found that both the BBU1 and CK treatments achieved the highest economic efficiency of the fertilizer regimes. Although the higher ratio of CRNF did not significantly increase total cost, it significantly decreased economic efficiency due to lower grain yields. These results indicate that grain yield, rather than fertilizer cost, is the primary factor influencing the economic benefit associated with one-time fertilizer application. However, there appears to be a contradiction between the yield and quality of rice, with increased N application between heading and maturity resulting in higher yields, but poor quality [48,59]. Therefore, the balance between yield and quality is likely to affect the adoption of one-time fertilization practices. The results of this study suggest that CRNF replacing CU at 50% total N helps to achieve a satisfactory balance between the yield and quality of direct-seeded rice under one-time fertilization, while obtaining high economic benefits.

## 4. Materials and Methods

### 4.1. Experimental Location and Materials

**Figure 4:**
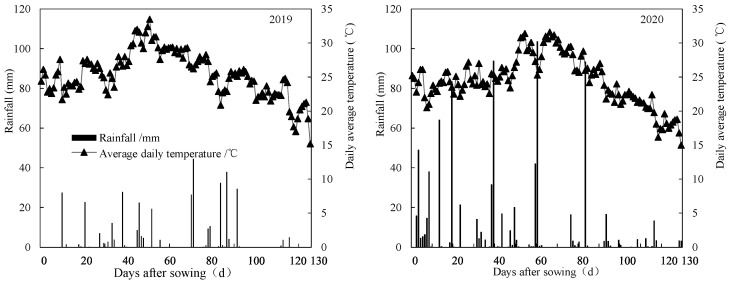
Rainfall and daily average temperatures during the rice-growing period in 2019 and 2020.

The field experiments were conducted in 2019 and 2020 in Shengao Town (34°54′ N, 120°21′ E), Jiangsu Province, China. This experiment site was within the rice-wheat crop rotation area in the middle and lower reaches of the Yangtze River, having a subtropical climate. The average of annual rainfall and temperature in this area is 1185.7 mm and 16.7 °C, respectively. The daily average temperature and rainfall during the growing season of rice measured at a weather station close to the experimental site are shown in Figure 4. The field soil was a clay loam with 31.72 g kg^−1^ organic matter, 1.82 g kg^−1^ total N, 139.0 mg kg^−1^ alkaline hydrolysable N, 37.81 mg kg^−1^ Olsen-P and 146.48 mg kg^−1^ exchangeable K in the topsoil (0–20 cm).

The rice variety ‘Nanjing 9108′ was selected as the material for this experiment. The CRNF (43.5% N) was a polymer-coated urea provided by Maoshi Ecological Fertilizer Co., Ltd. The conventional urea (CU, 46% N), calcium super-phosphate (12.5% P_2_O_5_), and potassium chloride (60% K_2_O) were sourced from nearby fertilizer sale points.

### 4.2. Experimental Design and Agricultural Practice

The field experiment included three one-time fertilization treatments (BBU1, BBU2, and BBU3) with different ratios of CRNF to CU, and split fertilization treatment as a control. We conducted a total of 4 treatments, each having 3 plots, and each plot being 50 m^2^ (5 m × 10 m). Mechanical dryland strip sowing was performed with a high-quality integrated planter machine (2BFMZ-350). This machine can sequentially perform basal fertilization, biaxial rotary tillage (matching 220 horsepower, tillage depth of approximately 15–18 cm) with straw burial, weed suppression in open seed rows, controlled depth sowing (depth of 2 cm, row spacing of 25 cm), rotary tillage mulching (depth of 0.7–1.0 cm), and compact suppression in a one-time operation. The nitrogen application rate and time of each treatment were shown in Table 6. Phosphate (130 kg ha^−1^ P_2_O_5_) and potassium fertilizers (150 kg ha^−1^ K_2_O) were applied on the day before mechanical operation. Rice seed was sown on 8 June 2019, and 10 June 2020, with a straw return rate of approximately 6.8 t ha^−1^. Rice seed was sown at a rate of 105 kg ha^−1^. After sowing, the rice field was filled with enough water to keep the field wet. The rice field remained flooded from initial tillering until the middle tillering stage, and the mid-term drainage was carried out from the mid-tillering to the panicle initiation stages. After that, the flooding state was maintained and the pre-harvest drainage was carried out 7 days before harvest. The management of pests, pathogens, and weeds was controlled by local methods, and all treatments were consistent. Harvesting was finalized on 25 October 2019, and 26 October 2020, respectively.

### 4.3. Sampling and Measurements of CRNF

The release profile of the CRNF was determined in 2020 using the buried-bag method [60]. Briefly, 10 g samples of CRNF were bagged and buried in the soil (5 cm deep and 20 cm apart) after rice sowing. In order to avoid the effect of CRNF samples on rice growth in the plot, the samples were buried in adjacent unfertilized plots. Three bags were collected every 10 days after burial. After rinsing the impurities on the surface of the mesh bag with flowing pure water, the nitrogen residual rate in the CRNF sample was determined using the Kjeldahl method [61]. The time interval nitrogen-release rate was expressed as the difference between the nitrogen residue rates at two adjacent measurement times.

### 4.4. Sampling and Measurements of Plants

At the panicle initiation, heading time, and maturity, the leaf area index and dry matter of 20 representative rice plants were measured and repeated three times. Then the photosynthetic potential and crop growth rate were calculated according to the following formulas [19]:

Photosynthetic potential (m^2^ d^−1^ m^−2^) = 1/2 × (L_1_ + L_2_)/(t_2_ − t_1_)

Crop growth rate (g m^−2^ d^−1^) = (W_2_ − W_1_)/(t_2_ − t_1_)

where t_1_ and t_2_ represent the days after sowing of twice measurements, L_1_ and L_2_ represent the leaf area (m^2^ m^−2^) measured at t_2_ and t_1_, respectively, W_1_ and W_2_ represent the shoots dry weight (g m^−2^) measured at t_1_ and t_2_, respectively.

After that, the plant samples were killed at 105 °C, dried at 80 °C until reaching a constant weight, and the dry weight of the samples was recorded. N content of the plants was determined using the Kjeldahl method [61] with crushed samples. N accumulation of plant biomass was considered as the product of its nitrogen content and dry weight. At maturity, 10 m^2^ rice grains were harvested in each plot and water content were measured. The final yield was expressed as the grain weight with 14.5% water content.

### 4.5. Measurements of Rice Quality

Brown rice, milled rice, and head rice were obtained from 150 g grain samples (3 replicates per treatment) using a shelling machine (SY88-TH, Double Dragon Group, Seoul, Republic of Korea), polishing machine (Pearlest, Kett Co., Ltd., Tokyo, Japan), and manual selection, respectively. After that, their rate to total grain weight (150 g) could be calculated. The chalky properties of milled rice were measured with a Wanshen SC-E analyzer for rice-appearance quality. The protein and amylose contents of milled rice were determined according to the Chinese national standards for good-quality rice (GB/T17891-2017). The protein components of milled rice were separated prior to the determination of albumin, globulin, glutelin, and prolamin contents using the method proposed in reference [38]. A Satake Rice Taste Analyzer (STA1A, Satake Co., Ltd., Tokyo, Japan) was used to assess the taste parameters of the milled rice, including its appearance, hardness, viscosity, balance value, and taste value.

### 4.6. Calculation of Rice Economic Benefit

The economic benefit associated with each treatment was determined according to the following formula:

(1) $$\mathrm{Economic}\;\mathrm{benefit}\;\left({\mathrm{CNY}\;\mathrm{ha}}^{-1}\right)=\mathrm{Rice}\;\mathrm{income}-\mathrm{Agricultural}\;\mathrm{inputs}\;\cos t$$

Rice income is calculated based on the current local grain price and grain yield. The local rice grain prices were 2500 CHY t^−1^ and 2550 CHY t^−1^ in 2019 and 2020, respectively. The agricultural inputs cost includes costs associated with materials (e.g., fertilizers, pesticides, and seeds) and mechanical operations (e.g., tillage, sowing, fertilization, pesticide spraying, irrigation, and harvesting).

### 4.7. Statistical Analysis

Microsoft Excel 2010 and SPSS 18.0 were used for data analyses, graphics, and statistical calculations. The significance analysis of Treatment (T), year (Y), and T×Y were based on two-year data. A 0.05 probability level was adopted for the least-significant difference test.

## 5. Conclusions

One-time fertilization did not significantly increase the grain yield or economic efficiency of direct-seeded rice, but significantly reduced rice chalkiness and improved eating quality, compared to split fertilization. In particular, CRNF replacing CU with 50% total N was found to increase grain yield by improving N accumulation, photosynthetic potential, and crop growth rate from heading to maturity under one-time fertilization. In addition, CRNF replacing conventional urea with 50% total N yielded increased economic benefits while reducing the frequency of fertilizer application. These results suggest that under the condition of replacing CU with CRNF in 50% total N, optimizing CRNF to obtain better yield and economic benefits is helpful to further improve the effect of one-time fertilization of direct-seeded rice.

## Figures and Tables

**Table 1:** Effects of different treatments on the photosynthetic potentials and crop growth rates.

Year	Treatment	Photosynthetic Potential (m^2^ d/m^2^)			Crop Growth Rate [g/(m^2^·d)]		
		ST-PI	PI-MT	HT-MA	ST-PI	PI-MT	HT-MA
2019	BBU1	136.1 a	163.4 a	318.8 a	8.6 a	28.1 b	14.5 a
	BBU2	125.5 b	151.5 b	288.5 b	7.6 b	26.2 c	13.1 b
	BBU3	123.6 b	146.3 b	276.5 c	7.3 b	25.3 c	12.4 b
	CK	116.9 c	145.7 b	292.1 b	6.7 c	30.5 a	14.1 a
2020	BBU1	129.2 a	153.2 a	294.2 a	9.1 a	29.3 b	15.4 a
	BBU2	118.5 b	142.7 ab	271.8 b	8.4 b	26.5 c	14.3 bc
	BBU3	113.5 bc	136.3 b	260.7 c	8.0 bc	25.9 c	13.8 c
	CK	108.7 c	138.1 b	277.8 b	7.8 c	31.1 a	14.7 ab
Year (Y)		**	**	**	**	*	**
Treatment (T)		**	**	**	**	**	**
Y × T		NS	NS	NS	NS	NS	NS

BBU1, both controlled-release nitrogen fertilizer (CRNF) and conventional urea (CU) were base-applied at a rate of 50% N each; BBU2, CRNF and CU were base-applied at a rate of 60% N and 40% N, respectively; BBU3, CRNF and CU were base-applied at a rate of 70% N and 30% N, respectively; CK, split application of CU. ST, PI, HT, and MA indicate sowing time, panicle initiation, heading time and maturity, respectively. The significance levels were as follows: NS, non-significant; *, *p* < 0.05; **, *p* < 0.01. Different lowercase letters after the data in the table indicate statistically significance within the same column and the same year (*p* < 0.05).

**Table 2:** Effects of different treatments on the milling quality and appearance quality of rice.

Year	Treatment	Milling Quality (%)			Appearance Quality (%)		
		Brown Rice Rate	Milled Rice Rate	Head Rice Rate	Chalky Kernel Rate	Chalky Area	Chalkiness
2019	BBU1	85.4	73.3	71.3	28.1 b	16.4 ab	4.6 b
	BBU2	84.9	72.6	70.8	27.0 b	15.7 b	4.2 b
	BBU3	85.0	72.5	70.2	24.3 c	14.0 c	3.4 c
	CK	84.3	72.3	69.3	34.1 a	17.8 a	6.0 a
2020	BBU1	84.7	74.0	71.5	28.2 b	14.8 b	4.2 b
	BBU2	84.4	73.8	71.0	27.5 b	14.5 bc	4.0 bc
	BBU3	84.5	73.4	71.4	27.2 b	12.9 c	3.5 c
	CK	84.1	73.2	70.3	35.2 a	18 a	6.3 a
Year (Y)		NS	NS	NS	NS	NS	NS
Treatment (T)		NS	NS	NS	**	**	**
Y × T		NS	NS	NS	NS	NS	NS

BBU1, both controlled-release nitrogen fertilizer (CRNF) and conventional urea (CU) were base-applied at 50% of total N each; BBU2, CRNF and CU were base-applied at a 60% and 40% of total N, respectively; BBU3, CRNF and CU were base-applied at 70% and 30% of total N, respectively; CK, split application of CU. The significance levels were as follows: NS, non-significant; **, *p* < 0.01. Different lowercase letters after the data in the table indicate statistically significance within the same column and the same year (*p* < 0.05).

**Table 3:** Effects of different treatments on the contents of amylose, protein, and its components in milled rice.

Year	Treatment	Amylose (%)	Protein (%)	Protein Components			
				Albumin (%)	Globulin (%)	Gliadin (%)	Glutenin (%)
2019	BBU1	11.3 b	9.5 ab	0.79 a	0.76 ab	0.98 b	6.22 ab
	BBU2	11.8 ab	9.1 b	0.71 b	0.70 bc	0.95 bc	5.81 bc
	BBU3	12.7 a	8.8 b	0.67 b	0.69 c	0.91 c	5.78 c
	CK	11.2 b	9.9 a	0.80 a	0.78 a	1.08 a	6.34 a
2020	BBU1	10.5 c	9.1 a	0.73 a	0.72 a	0.88 b	6.03 a
	BBU2	11.3 b	8.5 ab	0.67 ab	0.67 b	0.86 b	5.79 ab
	BBU3	11.9 a	8.3 b	0.67 b	0.63 b	0.84 b	5.35 b
	CK	10.4 c	9.2 a	0.75 a	0.74 a	0.96 a	6.16 a
Year (Y)		*	*	NS	NS	*	*
Treatment (T)		**	*	**	**	**	**
Y × T		NS	NS	NS	NS	NS	NS

BBU1, both controlled-release nitrogen fertilizer (CRNF) and conventional urea (CU) were base-applied at 50% of total N each; BBU2, CRNF and CU were base-applied at a 60% and 40% of total N, respectively; BBU3, CRNF and CU were base-applied at 70% and 30% of total N, respectively; CK, split application of CU. The significance levels were as follows: NS, non-significant; *, *p* < 0.05; **, *p* < 0.01. Different lowercase letters after the data in the table indicate statistically significance within the same column and the same year (*p* < 0.05).

**Table 4:** Effects of different treatments on the eating quality of milled rice.

Year	Treatment	Taste Value	Taste Value Parameters			
			Appearance	Hardness	Viscosity	Quality Of Balance
2019	BBU1	65.5 b	6.0 bc	7.3 ab	5.9 b	5.9 b
	BBU2	70.0 ab	6.6 ab	7.0 ab	6.8 a	6.6 a
	BBU3	71.5 a	7.0 a	6.9 b	7.2 a	6.9 a
	CK	60.5 c	5.3 c	7.6 a	5.1 c	5.1 c
2020	BBU1	68.8 b	6.3 b	7.6 ab	6.3 c	6.2 bc
	BBU2	74.0 a	7.1 a	7.0 c	7.2 ab	7.2 a
	BBU3	74.5 a	7.2 a	6.9 c	7.4 a	7.3 a
	CK	63.5 c	6.1 b	7.8 a	5.6 d	5.8 c
Year (Y)		*	NS	NS	NS	*
Treatment (T)		**	**	**	**	**
Y × T		NS	NS	NS	NS	NS

BBU1, both controlled-release nitrogen fertilizer (CRNF) and conventional urea (CU) were base-applied at 50% of total N each; BBU2, CRNF and CU were base-applied at a 60% and 40% of total N, respectively; BBU3, CRNF and CU were base-applied at 70% and 30% of total N, respectively; CK, split application of CU. The significance levels were as follows: NS, non-significant; *, *p* < 0.05; **, *p* < 0.01. Different lowercase letters after the data in the table indicate statistically significance within the same column and the same year (*p*< 0.05).

**Table 5:** Costs, income and economic benefit under different treatments (CNY ha^−1^).

Agricultural Items		2019				2020			
		BBU1	BBU2	BBU3	CK	BBU1	BBU2	BBU3	CK
**Costs**	Seeds	641	641	641	641	641	641	641	641
	N fertilizer	992	1064	1135	635	992	1064	1135	635
	P fertilizer	1144	1144	1144	1144	1144	1144	1144	1144
	K fertilizer	1250	1250	1250	1250	1250	1250	1250	1250
	Insecticides	150	150	150	150	150	150	150	150
	Herbicides	289	289	289	289	289	289	289	289
	Fungicides	1418	1418	1418	1418	1418	1418	1418	1418
	Tillaging and seeding	450	450	450	450	450	450	450	450
	Top-dressing	-	-	-	205	-	-	-	205
	Pesticide application	74	74	74	74	74	74	74	74
	Irrigation	570	570	570	570	570	570	570	570
	Mechanized harvesting	648	648	648	648	648	648	648	648
	Total	7625 a	7696 a	7768 a	7472 a	7625 a	7696 a	7768 a	7472 a
Income	Grains	25,000 a	23,250 b	22,750 b	24,750 a	24,225 a	22,440 b	21,930 b	23,715 a
Economic benefit		17,375 a	15,553 b	14,982 b	17,278 a	16,600 a	14,743 b	14,162 b	16,243 a

BBU1, both controlled-release nitrogen fertilizer (CRNF) and conventional urea (CU) were base-applied at 50% of total N each; BBU2, CRNF and CU were base-applied at a 60% and 40% of total N, respectively; BBU3, CRNF and CU were base-applied at 70% and 30% of total N, respectively; CK, split application of CU. Different lowercase letters after the data in the table indicate statistically significance within the same column and the same year (*p* < 0.05).

**Table 6:** Nitrogen (kg N ha^−1^) applied in each of the treatments and timing of application.

Treatment	Basal Fertilizer		Tillering Fertilizer (CU)	Spikelet-Promotion (CU)	Spikelet-Development (CU)
	CRNF	CU			
BBU1	135	135	0	0	0
BBU2	162	108	0	0	0
BBU3	189	81	0	0	0
CK	0	81	81	54	54

BBU1, both controlled-release nitrogen fertilizer (CRNF) and conventional urea (CU) were base-applied at 50% of total N each; BBU2, CRNF and CU were base-applied at a 60% and 40% of total N, respectively; BBU3, CRNF and CU were base-applied at 70% and 30% of total N, respectively; CK, split application of CU. Basal fertilizer was applied on the day before mechanical operation.; tillering fertilizer, spikelet-promotion fertilizer, and spikelet-development fertilizer were applied approximately 20 days, 55 days, and 65 days after sowing, respectively.

## Data Availability

All data are included in the present study.

## References

[1] Farooq M., Siddique K.H.M., Rehman H., Aziz T., Lee D.J., Wahid A. (2011). Rice direct seeding: Experiences, challenges and opportunities. Soil Tillage Res..

[2] Ehsanullah S.A.A., Umair A., Hamid R., Mohsin T., Imran K. (2014). Effect of sowing dates and weed control methods on weed infestation, growth and yield of direct-seeded rice. Philipp. Agric. Sci..

[3] Liu H.Y., Hussain S., Zheng M.M., Peng S.B., Huang J.L., Cui K.H., Nie L.X. (2015). Dry direct-seeded rice as an alternative to transplanted-flooded rice in Central China. Agron. Sustain. Dev..

[4] Zhang Y.F., Liu J., Yuan W., Zhang R.H., Xi X.B. (2021). Multiple leveling for paddy field preparation with double axis rotary tillage accelerates rice growth and economic benefits. Agriculture.

[5] Huang Q.Y., Fan X.L., Tang S.H., Zhang M., Huang X., Yi Q., Pang Y.W., Huang J.F. (2019). Seasonal differences in N release dynamic of controlled-released urea in paddy field and its impact on the growth of rice under double rice cropping system. Soil Tillage Res..

[6] Chen Y., Li S.Y., Zhang Y.J., Li T.T., Ge H.M., Xia S.M., Gu J.F., Zhang H., Lu B., Wu X.X. (2019). Rice root morphological and physiological traits interaction with rhizosphere soil and its effect on methane emissions in paddy fields. Soil Biol. Biochem..

[7] Fan M.S., Lu S.H., Jiang R.F., Liu X.J., Zhang F.S. (2009). Triangular transplanting pattern and split nitrogen fertilizer application increase rice yield and nitrogen fertilizer recovery. Agron. J..

[8] Peng S.B., Buresh R.J., Huang J.I., Zhong X.H., Zou Y.B., Yang J.C., Wang G.H., Liu Y.Y., Hu R., Tang Q.Y. (2010). Improving nitrogen fertilization in rice by sitespeciflc N management. A review. Agron. Sustain. Dev..

[9] Zhang H., Hou D.P., Peng X.L., Ma B.J., Shao S.M., Jing W.J., Gu J.F., Liu L.J., Wang Z.Q., Liu Y.Y. (2019). Optimizing integrative cultivation management improves grain quality while increasing yield and nitrogen use efficiency in rice. J. Integr. Agric..

[10] Zhou T.Y., Li Z.K., Li E.P., Wang W.L., Yuan L.M., Zhang H., Liu L.J., Wang Z.Q., Gu J.F., Yang J.C. (2022). Optimization of nitrogen fertilization improves rice quality by affecting the structure and physicochemical properties of starch at high yield levels. J. Integr. Agric..

[11] Yang Z.Y., Zhu Y.M., Zhang J.Y., Li X.Y., Ma P., Sun J.W., Sun Y.J., Ma J., Li N. (2022). Comparison of energy use between fully mechanized and semi-mechanized rice production in Southwest China. Energy.

[12] Yu F.H., Bai J.C., Jin Z.Y., Zhang H.G., Guo Z.H., Chen C.L. (2022). Research on precise fertilization method of rice tillering stage based on UAV hyperspectral remote sensing prescription map. Agronomy.

[13] Guo C., Ren T., Li P.F., Wang B., Zou J.L., Hussain S., Cong R.H., Wu L.S., Lu J.W., Li X.K. (2019). Producing more grain yield of rice with less ammonia volatilization and greenhouse gases emission using slow/controlled-release urea. Environ. Sci. Pollut. Res. Inter..

[14] Chu G., Chen T.T., Chen S., Xu C.M., Zhang X.F., Wang D.Y. (2018). Polymer-coated urea application could produce more grain yield in “super” rice. Agron. J..

[15] Wu Q., Wang Y., Ding Y., Tao W., Gao S., Li Q., Li W., Liu Z., Li G. (2021). Effects of different types of slow-and controlled-release fertilizers on rice yield. J. Integr. Agric..

[16] Yang X., Tan X.L., Wu L.H. (2021). Grain yield and nitrogen use efficiency response to polymer coated urea for two indica-japonica hybrid rice varieties. J. Plant Nutr..

[17] Wang L., Xue C., Pan X., Chen F., Liu Y. (2018). Application of Controlled-Release urea enhances grain yield and nitrogen use efficiency in irrigated rice in the Yangtze River basin, China. Front. Plant Sci..

[18] Tian C., Sun M., Zhou X., Li J., Xie G., Yang X., Peng J. (2022). Increase in yield and nitrogen use efficiency of double rice with long-term application of controlled-release urea. J. Integr. Agric..

[19] Xu D., Zhu Y., Zhu H.B., Hu Q., Liu G.D., Wei H.Y., Zhang H.C. (2021). Effects of a one-time application of Controlled-Release nitrogen fertilizer on yield and nitrogen accumulation and utilization of late japonica rice in China. Agriculture.

[20] Cheng S., Xing Z.P., Tian C., Li S.P., Tian J.Y., Liu Q.Y., Hu Y.J., Guo B.W., Hu Q., Wei H.Y. (2022). Effects of controlled release urea formula and conventional urea ratio on grain yield and nitrogen use efficiency of Direct-Seeded rice. Agriculture.

[21] Pan S.G., Wen X.C., Wang Z.M., Ashraf U., Tian H., Duan M.Y., Mo Z.W., Fan P.S., Tang X.R. (2017). Benefits of mechanized deep placement of nitrogen fertilizer in direct-seeded rice in South China. Field Crop. Res..

[22] Xing Z.P., Hu Y.J., Qian H.J., Cao W.W., Guo B.W., Wei H.Y., Xu K., Huo Z.Y., Zhou G.S., Dai Q.G. (2017). Comparison of yield traits in rice among three mechanized planting methods in a rice-wheat rotation system. J. Integr. Agric..

[23] Scott J., Stephen B. (2020). The spread of smaller engines and markets in machinery services in rural areas of South Asia. J. Rural Stud..

[24] Zhang J., Zhang Y., Song N., Chen Q., Sun H., Peng T., Huang S., Zhao Q. (2021). Response of grain-filling rate and grain quality of mid-season indica rice to nitrogen application. J. Integr. Agric..

[25] Chu G., Wang Z.Z., Zhang H., Liu L.J., Yang J.C., Zhang J.H. (2015). Alternate wetting and moderate drying increases rice yield and reduces methane emission in paddy field with wheat straw residue incorporation. Food Energy Secur..

[26] Kim S.Y., Gutierrez J., Kim P.J. (2016). Unexpected stimulation of CH_4_ emissions under continuous no-tillage system in mono-rice paddy soils during cultivation. Geoderma.

[27] Wang Y.J., Huang G.Q. (2022). A two-step framework for dispatching shared agricultural machinery with time windows. Comput. Electron. Agric..

[28] Wang Y.J., Qiao J.Y., Ji W.Y., Sun J., Huo D.X., Liu Y.P., Chen H.T. (2022). Effects of crop residue managements and tillage practices on variations of soil penetration resistance in sloping farmland of Mollisols. Int. J. Agric. Biol. Eng..

[29] Tang J.C., Zhang R.Y., Li H.C., Zhang J., Chen S.Q., Lu B.L. (2020). Effect of the applied fertilization method under full straw return on the growth of mechanically transplanted rice. Plants.

[30] Weng W.A., Cheng S., Li S.P., Tian J.Y., Tao Y., Hu Q., Hu Y.J., Guo B.W., Wei H.Y., Xing Z.P. (2021). Effects of one-off nitrogen basal fertilization on yield of direct seeding conventional japonicarice under different panicle formation types. J. Agric. Sci. Technol..

[31] Cheng S., Li S.P., Tian J.Y., Xing Z.P., Hu Y.J., Guo B.W., Wei H.Y., Gao H., Zhang H.C. (2020). Effects of one-time nitrogen basal application on the yield and quality of different direct-seeding rice crops by machine. Trans. Chin. Soc. Agric. Eng..

[32] Baggs E.M., Stevenson M., Pihlatie M., Regar A., Cook H., Cadisch G. (2003). Nitrous oxide emissions following application of residues and fertiliser under zero and conventional tillage. Plant Soil..

[33] Garnett T., Conn V., Kaiser B.N. (2009). Root based approaches to improving nitrogen use efficiency in plants. Plant Cell Environ..

[34] Liu T.Q., Fan D.J., Zhang X.X., Chen J., Li A., Cao C.G. (2015). Deep placement of nitrogen fertilizers reduces ammonia volatilization and increases nitrogen utilization efficiency in no-tillage paddy fields in central China. Field Crop. Res..

[35] Chen Z.M., Wang Q., Ma J.C., Zhao J., Huai Y., Ma J.W., Ye J., Yu Q.G., Zou P., Sun W.C. (2022). Combing mechanical side-deep fertilization and controlled-release nitrogen fertilizer to increase nitrogen use efficiency by reducing ammonia volatilization in a double rice cropping system. Front. Environ. Sci..

[36] Zhang F.S., Cui Z.L., Fan M.S., Zhang W.F., Chen X.P., Jiang R.F. (2011). Integrated soil-crop system management: Reducing environmental risk while increasing crop productivity and improving nutrient use efficiency in China. J. Environ. Qual..

[37] Chen Y.T., Peng J., Wang J., Fu J., Hou Y., Zhang C.D., Fahad S., Peng S.B., Cui K.H., Nie L.X. (2015). Crop management based on multi-split topdressing enhances grain yield and nitrogen use efficiency in irrigated rice in China. Field Crops Res..

[38] Wei H.Y., Chen Z.F., Xing Z.P., Zhou L., Liu Q.Y., Zhang Z.Z., Jiang Y., Hu Y., Zhu J., Cui P.Y. (2018). Effects of slow or controlled release fertilizer types and fertilization modes on yield and quality of rice. J. Integr. Agric..

[39] Lyu Y.F., Yang X.D., Pan H.Y., Zhang X.H., Cao H.X., Ulgiati S., Wu J., Zhang Y.Z., Wang G.Y., Xiao Y.L. (2021). Impact of fertilization schemes with different ratios of urea to controlled release nitrogen fertilizer on environmental sustainability, nitrogen use efficiency and economic benefit of rice production: A study case from Southwest China. J. Clean. Prod..

[40] Golden B.R., Slaton N.A., Norman R.J., Wilson C.E., Delong R.E. (2009). Evaluation of polymer-coated urea for direct-seeded, delayed-flood rice production. Soil Sci. Soc. Am. J..

[41] Timilsena Y.P., Adhikari R., Casey P., Muster T., Gill H., Adhikari B. (2015). Enhanced efficiency fertilisers: A review of formulation and nutrient release patterns. J. Sci. Food Agric..

[42] Diana F., Mohamed O., Abdelaziz T., Jean-Paul G. (1997). Molecular records of micro-evolution within the Algerian population of *Fusarium oxysporum* f. sp. *albedinis* during its spread to new oases. Eur. J. Plant Pathol..

[43] Kakar K., Nitta Y., Asagi N., Komatsuzaki M., Shiotsu F., Kokubo T., Xuan T.D. (2019). Morphological analysis on comparison of organic and chemical fertilizers on grain quality of rice at different planting densities. Plant Prod. Sci..

[44] You H., Zafar S., Zhang F., Zhu S.B., Chen K., Shen C.C., Zhao X.Q., Zhang W.Z., Xu J.L. (2022). Genetic mechanism of heterosis for rice milling and appearance quality in an elite rice hybrid. Crop J..

[45] Grigg B.C., Siebenmorgen T.J., Norman R.J. (2016). Effects of nitrogen rate and harvest moisture content on physicochemical properties and milling yields of rice. Cereal Chem..

[46] Yoshida H., Takehisa K., Kojima T., Ohno H., Sasaki K., Nakagawa H. (2016). Modeling the effects of N application on growth, yield and plant properties associated with the occurrence of chalky grains of rice. Plant Prod. Sci..

[47] Bahuguna R.N., Solis C.A., Shi W., Jagadish K.S.V. (2017). Post-flowering night respiration and altered sink activity account for high night temperature-induced grain yield and quality loss in rice (*Oryza sativa* L.). Physiol. Plant..

[48] Wei H.H., Ge J.L., Zhang X.B., Zhu W., Deng F., Ren W.J., Chen Y.L., Meng T.Y., Dai Q.G. (2022). Decreased panicle N application alleviates negative effects of shading on rice grain yield and grain quality. J. Integr. Agric..

[49] Chen Y., Wang M., Ouwerkerk P.B.F. (2012). Molecular and environmental factors determining grain quality in rice. Food Energy Secur..

[50] Toutounji M.R., Farahnaky A., Santhakumar A.B., Oli P., Butardo V.M., Blanchard C.L. (2019). Intrinsic and extrinsic factors affecting rice starch digestibility. Trends Food Sci. Technol..

[51] Balindong J.L., Ward R.M., Liu L., Rose T.J., Pallas L.A., Ovenden B.W., Snell P.J., Waters D.L.E. (2018). Rice grain protein composition influences instrumental measures of rice cooking and eating quality. J. Cereal Sci..

[52] Amardeep S.V., Narpinder S., Priyanka P., Parmeet K., Amritpal K. (2019). Evaluation of head and broken rice of long grain *Indica* rice cultivars: Evidence for the role of starch and protein composition to head rice recovery. Food Res. Int..

[53] Ning H.F., Qiao J.F., Liu Z.H., Lin Z.M., Li G.H., Wang Q.S., Wang S.H., Ding Y.F. (2010). Distribution of proteins and amino acids in milled and brown rice as affected by nitrogen fertilization and genotype. J. Cereal Sci..

[54] Martin M., Fitzgerald M.A. (2001). Proteins in rice grains influence cooking properties!. J. Cereal Sci..

[55] Wang C.L., Zhang Y.D., Zhu Z., Chen T., Zhao Q.Y., Zhong W.G., Yang J., Yao S., Zhou L.H., Zhao L. (2017). Integrative agriculture; findings from Jiangsu academy of agricultural science broaden understanding of integrative agriculture. Agric. Week..

[56] Bian J.L., Xu F.F., Han C., Qiu S., Ge J.L., Xu J., Zhang H.C., Wei H.Y. (2018). Effects of planting methods on yield and quality of different types of japonica rice in northern Jiangsu plain, China. J. Integr. Agric..

[57] Gao J., Xu C., Luo N., Liu X., Huang S., Wang P. (2021). Mitigating global warming potential while coordinating economic benefits by optimizing irrigation managements in maize production. J. Environ. Manag..

[58] Kumar A., Rana K.S., Choudhary A.K., Bana R.S., Sharma V.K., Prasad S., Gupta G., Choudhary M., Pradhan A., Rajpoot S.K. (2021). Energy budgeting and carbon footprints of zero-tilled pigeonpea–wheat cropping system under sole or dual crop basis residue mulching and Zn-fertilization in a semi-arid agro-ecology. Energy.

[59] Cheng B., Jiang Y., Cao C.G. (2021). Balance rice yield and eating quality by changing the traditional nitrogen management for sustainable production in China. J. Clean. Prod..

[60] Ke J., He R.C., Hou J.H., Ding C., Ding Y.F., Wang S.H., Liu Z.H., Tang S., Ding C.Q., Chen L. (2018). Combined controlled-released nitrogen fertilizers and deep placement effects of N leaching, rice yield and N recovery in machine-transplanted rice. Agric. Ecosyst. Environ..

[61] Bremner J.M. (1960). Determination of nitrogen in soil by the Kjeldahl method. J. Agric. Sci..

